# Influence of Plant Age and Endophyte Status on the Nematotoxicity of *Festulolium loliaceum* to *Trichodorus primitivus* and Quantification of Active Phytochemicals

**DOI:** 10.3390/toxins18030125

**Published:** 2026-03-01

**Authors:** Nyambura G. Mwangi, Timothy J. Gillanders, Mark Stevens, Alistair J. D. Wright, Simon G. Edwards, Martin C. Hare, Matthew A. Back

**Affiliations:** 1 Agriculture and Environment Department, Harper Adams University, Newport TF10 8NB, UK; 2 Cropmark Seeds Ltd., 49 Manion Road, RD7, Christchurch 7677, New Zealand; 3 British Beet Research Organisation, Centrum, Norwich Research Park, Colney Lane, Norwich NR4 7UG, UK

**Keywords:** *Festulolium* hybrids, grass endophytes, immobility, nematotoxic, lolines, flavonoids, phenols, mutualism

## Abstract

*Festulolium* hybrids are cool-season forage grasses that form symbiotic relationships with the fungus *Epichloë uncinata*, which produces loline alkaloids that protect the host from herbivores. This study evaluated the nematotoxicity of shoot and root extracts of *Festulolium loliaceum* against the stubby root nematode *Trichodorus primitivus*. Methanolic root and shoot extracts from plants aged 8, 12, 16, and 20 weeks were tested in vitro at five concentrations (312.5–5000 µg mL^−1^) over 24, 48, and 72 h. Nematode immobility, mortality, and phytochemical profiles, including flavonoids, loline alkaloids, and phenols, were quantified. Extracts from shoots caused significant concentration and time-dependent immobility of *T. primitivus* (*p* = 0.001), reaching ≥90% at 5000 µg mL^−1^ after 72 h in 8–12-week-old plants. Endophyte presence enhanced nematotoxicity, where LD_50_ values for E+ roots were two-fold lower at 12 weeks and fifty-fold lower at 20 weeks compared with E− root extracts. Shoot extracts of E+ grass had the highest nematicidal activity at 8 weeks, with a significantly lower LD_50_ value than E− (*p* < 0.05). Loline alkaloid concentrations increased with plant age, while flavonoids and phenols declined. Nematotoxicity of *F. loliaceum* extracts was strongly influenced by plant age and endophyte presence. Younger E+ shoots produced the most potent shoot extracts, whereas older plants produced the most potent root extracts. Flavonoid content was negatively correlated with shoot biomass (R = −0.94, *p* < 0.001). Similarly, phenol content was negatively correlated to both root biomass (R = −0.79, *p* < 0.001) and shoot biomass (R = −0.67, *p* < 0.005).

## 1. Introduction

*Epichloë* clavicipitaceous fungal endophytes commonly establish symbiotic relationships with cool-season grasses. This association results in the synthesis of defence secondary metabolites such as ergot and loline alkaloids [1,2]. This mutualistic association is highly intimate, as many alkaloids cannot be synthesised independently by either partner [3]. For instance, the fungal endophyte *Epichloë uncinata* in *Festuca–Lolium* hybrids used in this study must be present for the production of loline alkaloids [4,5]. The hybrid plants synthesise a suite of loline alkaloids, including *N*-acetylloline (NAL), *N*-methylloline (NML), *N*-formylnorloline (NFNL), norloline (NL), loline (L), and *N*-acetylnorloline (NANL), with NFL and NAL being the most abundant [5]. The loline alkaloid concentrations are highest in seeds and pseudo stems. Although the endophyte does not colonise roots, lolines are detected there due to xylem-mediated translocation from sites of synthesis [5].

The occurrence and concentration of these alkaloids are influenced by multiple factors, including host genotype and fungal strain [1,6]. Abiotic factors such as temperature and humidity influence the alkaloid levels. For instance, ergovaline and lolitrem B concentrations increase with higher summer temperatures and decline as temperatures lower [7]. The plant growth phases, such as reproduction and maturation, are known to elevate the concentrations [8], while peramine levels decline in the leaves as plants senesce [9]. The availability of nutrients further modifies alkaloid accumulation. Phosphorus levels between 17 and 50 mg kg^−1^ are known to increase ergot alkaloids in shoots, while 96 mg kg^−1^ decreases them; root concentrations, however, increase linearly with soil phosphorus [10].

In addition to alkaloids, these grasses contain other secondary metabolites such as phenolic compounds, flavonoids, amino acids, and sugars. Roots of tall fescue exude phenolic acids, including cinnamic, ferulic, gallic, gentisic, and syringic acids under hydroponic conditions [11]. Production of these compounds in endophyte-associated grasses has been linked to negative effects on soil microbes [12], and some, such as caffeoylquinic acid, isomers, and chlorogenic acid, have been shown to occur independently of the endophyte [13]. Phenolic compounds exhibit direct nematotoxicity [14] and can induce systemic resistance in plants [15,16], while certain flavonoids act as phytoalexins that enhance resistance to nematode infection [16,17].

Fungal endophyte strains such as the U2 strain of *E. uncinata* used in this study have been made commercially available, as they have been successfully used as biocontrol agents in pasture systems [5]. Both purified alkaloids and crude grass extracts from these grass-endophyte associations have the ability to cause repellent, nematicidal, and hatch-inhibiting impacts on nematodes [18]. Their efficacy depends on alkaloid class and concentration, exposure time, plant tissue, and plant age [19]. Pure NFL, for example, repels *Pratylenchus scribneri* in vitro [20]. Notably, in the absence of the endophyte, extracts from shoots and roots of E− grasses were reported to cause mortality to juveniles of *Meloidogyne incognita* [21]. Similar patterns occur in hybrid *Festulolium* spp., where *M. incognita* juveniles show reduced activity regardless of endophyte presence [5], suggesting that additional plant-derived compounds not associated with the endophyte also contribute to nematode suppression.

Stubby root nematodes such as *Trichodorus primitivus* (family Trichodoridae) are plant-parasitic ectoparasites that feed on root epidermal cells, causing a characteristic stubby root system [22]. This root deformation reduces water and nutrient uptake, leading to stunting and yield loss. In sugar beet, *T. primitivus* and related species cause docking disorder, resulting in up to 50% reductions in sugar yield [23,24]. In potatoes, they are important vectors of Tobacco Rattle Virus (TRV) [25,26,27,28].

Despite growing evidence of nematotoxicity in endophyte-colonised grasses across multiple nematode species [18,19,29], the relative contributions of plant age, endophyte status, and phytochemical composition to direct nematode suppression remain poorly understood. The specific secondary metabolites responsible for these effects are also not clearly defined. This study builds on a previous field experiment in which stubby root nematode reproduction was significantly lower in plots planted with endophyte-colonised *Festulolium* hybrids (E+) compared with endophyte-free (E−) grasses [24]. Under laboratory conditions, we evaluated how endophyte status influences the nematotoxicity of extracts obtained from roots and shoots on *T. primitivus.*

Specifically, the study assessed how endophyte status, plant age, extract concentration, and exposure time influence effects on nematode immobility and mortality and quantified the concentrations of key phytochemicals associated with these grasses. The study was based on the hypothesis that endophyte status and plant age significantly affect the nematotoxic potential of *F. loliaceum* extracts by altering the concentrations of loline alkaloids, phenolic compounds, and flavonoids. By integrating bioassays with phytochemical profiling, this work provides new insights into endophyte-mediated nematode suppression and highlights the potential of *Festulolium* hybrids as biologically active forages for sustainable nematode management.

## 2. Results

**Figure 1:**
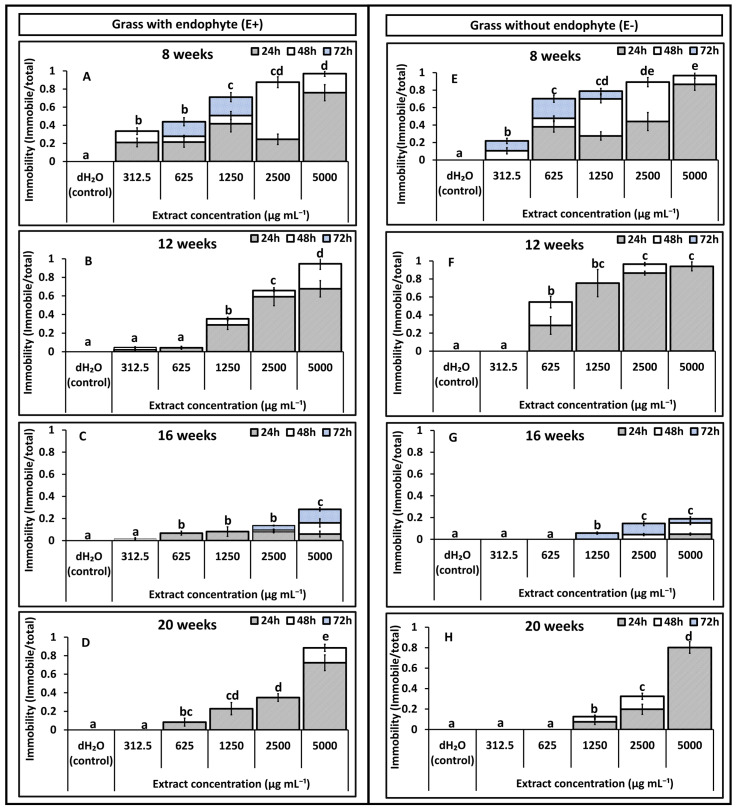
Experiment 1: Effects of *Festulolium loliaceum* shoot extracts, with (E+) and without endophyte (E−), on the immobility of *Trichodorus primitivus*. Panels (**A**–**D**) show responses to E+ extracts and panels (**E**–**H**) to E− extracts obtained from grass at 8, 12, 16, and 20 weeks of growth, respectively. Extract concentrations (*x*-axis) ranged from 312.5 to 5000 µg/mL, with distilled water (dH_2_O) as the control. Nematode immobility (*y*-axis) was monitored after 24, 48, and 72 h. Lowercase letters above the bars within each age represent statistical significance at (*p* < 0.05) in cumulative immobility after 72 h, according to multiple comparison tests. Standard error of the mean (SEM) is indicated by the error bars.

**Figure 2:**
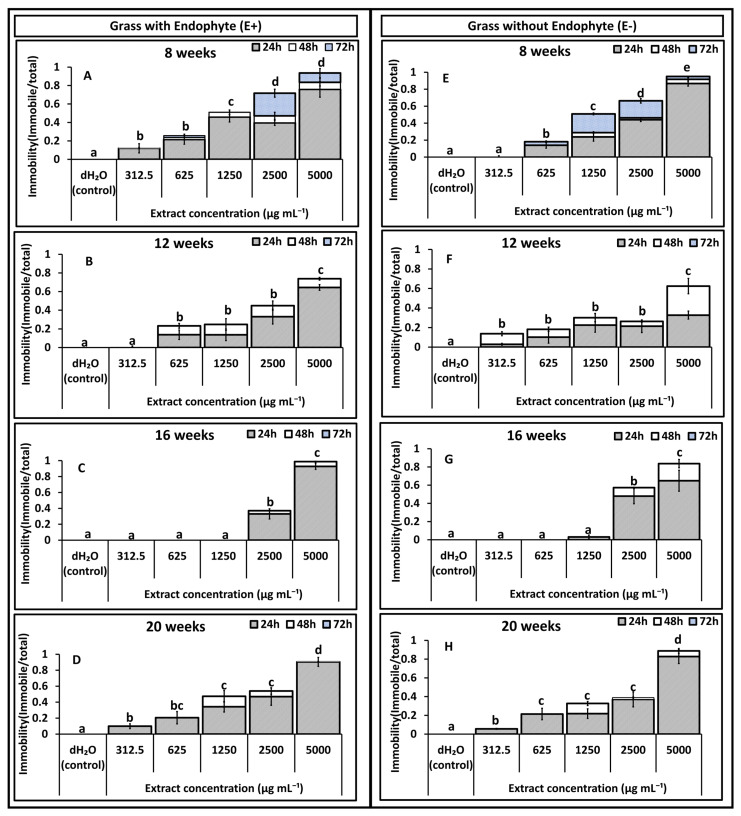
Experiment 2: Effects of *Festulolium loliaceum* shoot extracts, with (E+) and without endophyte (E−), on the immobility of *Trichodorus primitivus*. Panels (**A**–**D**) show responses to E+ extracts and panels (**E**–**H**) to E− extracts obtained from grass at 8, 12, 16, and 20 weeks of growth, respectively. Extract concentrations (*x*-axis) ranged from 312.5 to 5000 µg/mL, with distilled water (dH_2_O) as the control. Nematode immobility (*y*-axis) was monitored after 24, 48, and 72 h. Lowercase letters above the bars within each age represent statistical significance at (*p* < 0.05) in cumulative immobility after 72 h, according to multiple comparison tests. Standard error of the mean (SEM) is indicated by the error bars.

**Figure 3:**
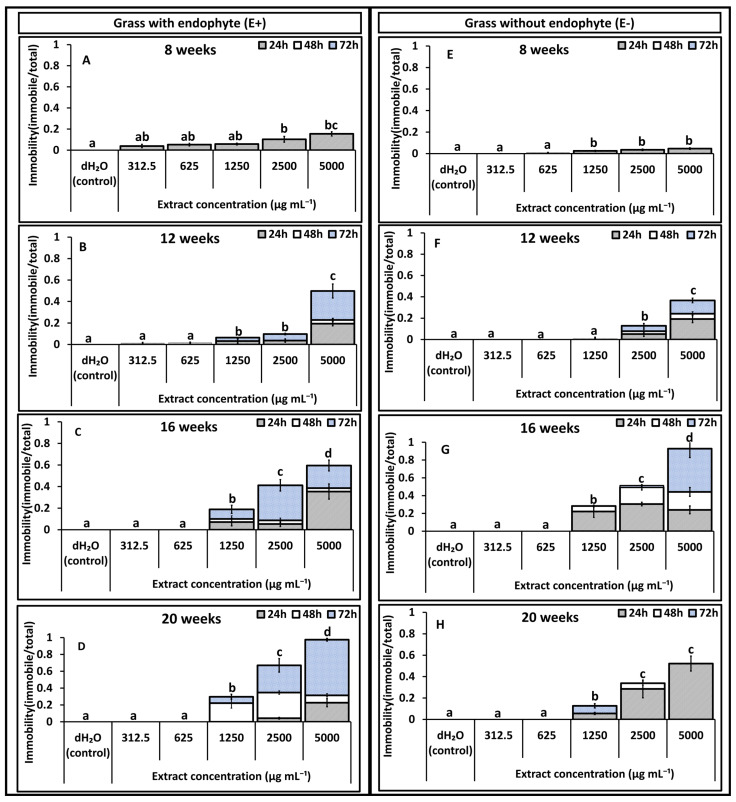
Experiment 1: Effects of *Festulolium loliaceum* root extracts, with (E+) and without endophyte (E−), on the immobility of *Trichodorus primitivus*. Panels (**A**–**D**) show responses to E+ extracts and panels (**E**–**H**) to E− extracts obtained from grass at 8, 12, 16, and 20 weeks of growth, respectively. Extract concentrations (*x*-axis) ranged from 312.5 to 5000 µg/mL, with distilled water (dH_2_O) as the control. Nematode immobility (*y*-axis) was monitored after 24, 48, and 72 h. Lowercase letters above the bars within each age represent statistical significance at (*p* < 0.05) in cumulative immobility after 72 h, according to multiple comparison tests. Standard error of the mean (SEM) is indicated by the error bars.

**Figure 4:**
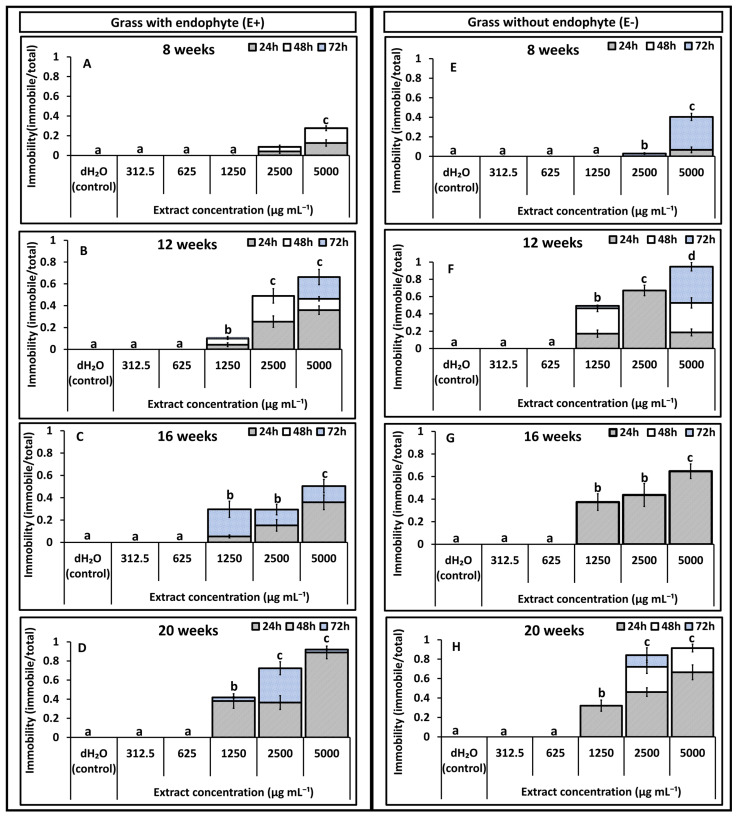
Experiment 2: Effects of *Festulolium loliaceum* root extracts, with (E+) and without endophyte (E−), on the immobility of *Trichodorus primitivus*. Panels (**A**–**D**) show responses to E+ extracts and panels (**E**–**H**) to E− extracts obtained from grass at 8, 12, 16, and 20 weeks of growth, respectively. Extract concentrations (*x*-axis) ranged from 312.5 to 5000 µg/mL, with distilled water (dH_2_O) as the control. Nematode immobility (*y*-axis) was monitored after 24, 48, and 72 h. Lowercase letters above the bars within each age represent statistical significance at (*p* < 0.05) in cumulative immobility after 72 h, according to multiple comparison tests. Standard error of the mean (SEM) is indicated by the error bars.

Results on immobility effects reported in Figure 1, Figure 2, Figure 3 and Figure 4 are pre-recovery measurements (24, 48, and 72 h) and therefore indicate temporary immobilisation potential, classified here as nematostatic effects. Mortality was assessed after a 48 h recovery period in distilled water and calculated as LD_50_ estimates, which quantify lethal (nematicidal) effects, reported in Table 1 and Table 2.

### 2.1. Effect of Shoot Extracts on T. primitivus Immobility

In Experiment 1, shoot extracts reduced nematode mobility in a concentration (*p* = 0.001) and time (*p* = 0.02) responsive manner across all ages (8–20 weeks), with effects weakest at 312.5 µg mL^−1^ (0–5%) and strongest at 5000 µg mL^−1^ after 72 h for both treatments (Figure 1). At 8 and 12 weeks (Figure 1A,B,E,F), both treatments (E+ and E−) produced high immobility, exceeding 90% at 5000 µg mL^−1^ after 72 h. At most ages, immobility at 312.5 and 625 µg mL^−1^ did not differ significantly from the control (H_2_O), except at 8 weeks (Figure 1A,E), where both E+ and E− extracts produced higher immobility.

Immobility increased with exposure time across all concentrations. Notably, at 5000 µg mL^−1^, more than 60% immobility was already evident within 24 h for most ages, except for 16-week extracts in E− and E+ extracts (Figure 1C,G). At lower concentrations, longer exposure times were required to achieve higher immobilisation. A significant decline in immobility (*p* = 0.0002) was recorded as plant age increased in shoots extracts for both treatments. An exception occurred in E+ extracts from 20-week-old plants, which showed a marked increase in immobility (Figure 1D) compared with the reduced activity observed at 16 weeks (Figure 1C). Significant interaction effects were detected for age × endophyte status (*p* = 0.01), concentration × endophyte status (*p* = 0.003), and age × concentration (*p* = 0.04), indicating that nematode immobilisation was jointly influenced by plant age, extract concentration, and endophyte status.

In experiment 2, a similar trend was observed. Nematode immobility declined post exposure to extracts from both treatments relative to the control (dH_2_O) across most concentrations and plant ages (Figure 2A–H). Immobilisation increased in a clear concentration-dependent manner for both extract types, with the highest concentration (5000 µg mL^−1^) producing the greatest immobility (62–95%) and the lowest concentration (312.5 µg mL^−1^) resulting in minimal immobility (0–9%). For E+ extracts (Figure 2A–D), immobility rose consistently with concentration at all plant ages. At the highest concentration, immobility was similar between the treatments except at 16 weeks (Figure 2C), where both treatments produced comparatively lower immobility. In E− extracts (Figure 2E–H), plant age strongly influenced activity: extracts from younger plants (8 and 12 weeks) caused higher nematode immobility at lower concentrations (312.5–1250 µg mL^−1^) than extracts from older plants (16 and 20 weeks; Figure 2G,H). Significant interaction effects were again detected between age × endophyte status (*p* = 0.011), endophyte status × extract concentration (*p* = 0.003), and age × extract concentration (*p* = 0.03), confirming that nematode immobilisation was jointly shaped by plant age, extract concentration, and endophyte status.

### 2.2. Root Extracts

Immobility caused by root extracts was generally lower in both treatments (E+ and E−) compared with those recorded in shoot extracts. In experiment 1, negligible (<5%) to zero immobility was observed at the lowest concentrations (312.5 and 625 µg mL^−1^) in E+ and E− extracts (Figure 3A–H). The immobility of *T. primitivus* increased progressively with extract concentration, exposure time, and plant age for both treatments (Figure 3B–D,F–H). Extracts from 8-week-old plants caused the lowest immobility in both treatments, particularly for E− grass, where immobility remained below 5% (Figure 3E). In comparison, E+ extracts from 8-week-old plants induced slightly higher immobility, ranging from 3 to 15% (Figure 3A). By 12 weeks, E+ extracts produced a clear response, with 55–65% immobility at 5000 µg mL^−1^ after 72 h exposure (Figure 3B), while E− extracts recorded 15–40% immobility at concentrations between 2500 and 5000 µg mL^−1^ (Figure 3F).

Immobility peaked between 16 and 20 weeks (Figure 3C–H). At 16 weeks, immobility ranged from 22 to 98% as concentration increased from 1250 to 5000 µg mL^−1^ after 72 h for E− extracts and from 16 to 57% for E+ extracts (Figure 3C,G). At 20 weeks, immobility in E− extracts declined to 52% at the highest concentration (5000 µg mL^−1^), while E+ extracts increased to 96% at the same concentration and exposure duration (Figure 3D,H). Significant interaction effects were detected for age × endophyte status (*p* = 0.01) and concentration of extract × endophyte status (*p* = 0.04), indicating that both plant age and extract concentration influenced nematode immobilisation differently between E+ and E− roots.

In the second in vitro bioassay using root extracts, higher immobility rates were recorded compared with the first experiment, although the overall patterns remained similar (Figure 4A–H). Younger root extracts caused lower *T. primitivus* immobility than older extracts for both treatments, while a clear dose-dependent effect was evident in all cases. Little to no immobility occurred at 312.5 and 625 µg mL^−1^ for either extract type. Immobility of 8-week-old roots remained low (0–5%) across 312.5–1250 µg mL^−1^, reaching only 35% at 5000 µg mL^−1^ (Figure 4A,E). By 12 weeks, immobility became more pronounced: at 5000 µg mL^−1^, E+ extracts achieved approximately 80% immobility by 72 h exposure (Figure 4B), while E− extracts reached 60%, driven mainly by 24–48 h exposure times (Figure 4F). At 20 weeks, the highest immobility was recorded, with both E+ and E− extracts achieving 100% immobility after 72 h exposure (Figure 4D,H). The only significant interaction effect detected was for endophyte status × age (*p* = 0.02), confirming that the influence of extract concentration on nematode immobility varied with both endophyte status and plant developmental stage.

### 2.3. Nematotoxic Effects of Extracts on T. primitivus

Overall, shoot extracts from grasses with endophyte (E+) were more nematotoxic compared with those from E-grasses, as indicated by significantly lower LD_50_ values across different ages and concentrations (Table 1). Despite the high immobility recorded in E− extracts, the effects were reversible, as evidenced by the high LD_50_ values obtained after nematodes were rinsed in distilled water. The LD_50_ values recorded for shoot extracts followed an age-dependent pattern, with the lowest values observed in younger plants and the highest in older plants for both E+ and E-grass. The only exception occurred at 20 weeks in E+ grasses, where the LD_50_ was significantly lower than that recorded at 16 weeks. For root extracts, the treatments followed different age-related trends: in E+ root extracts, LD_50_ values decreased with increasing age, while in E− roots, LD_50_ values increased as plants matured, where extracts from 8-week-old grass from both treatments had no effect on the nematodes (Table 1).

In 8-week-old shoot extracts, the LD_50_ was 627 µg mL^−1^ for E+ grasses compared with 860 µg mL^−1^ for E-grasses, translating to approximately 1.4-fold greater nematotoxicity in E+ grasses. The contrast between E+ and E-increased with plant age: at 12 and 20 weeks, the LD_50_ values from E+ shoot extracts were three-fold and seventeen-fold more nematotoxic, respectively, than those from E− extracts of comparable age. In root extracts, no nematotoxic effects were detected in either E+ or E− grasses at 8 weeks. By 12 weeks, E+ roots (3399 µg mL^−1^) were nearly twice as potent as E− roots (6471 µg mL^−1^). The contrast was most pronounced at 20 weeks, when E+ roots (2846.62 µg mL^−1^) were nearly 50 times more potent than E− roots (140,093 µg mL^−1^) (Table 1).

In the second experiment, similar patterns of LD_50_ variation with endophyte status and age were observed for both root and shoot extracts (Table 2). Highest nematicidal effects were still highest in E+ compared to E-, as indicated by consistently lower LD_50_ values across most plant ages. The nematotoxicity of shoot extracts from E+ grasses was approximately 1.2-, 1.5-, 2.5-, and 7.9-fold greater at 8, 12, 16, and 20 weeks, respectively, compared with E-grasses of the same age.

Like Experiment 1, shoot extracts from younger plants had the highest nematicidal activity compared to older plants for both treatments, with one exception: at 16 weeks, where E+ shoot extracts showed lower nematicidal activity than at 20 weeks. For root extracts, no nematicidal activity was observed in samples from 8-week-old plants for both treatments.

The nematotoxicity differences between E+ and E− extracts became more pronounced with plant age (Table 2); by 20 weeks, E+ root extracts were approximately six times more nematotoxic than E− extracts.

### 2.4. Phytochemical Analysis

#### 2.4.1. Loline Alkaloids

Loline alkaloids NFL, NAL, and NANL were assessed in both E+ and E− grass. Across all ages, E− grass extracts had no lolines. Plant age affected both the overall concentration and the individual composition of loline alkaloids, with tissues (roots and shoots) differing in their levels and alkaloid profiles. Total loline alkaloid concentrations increased progressively with plant age, such that at 20 weeks the total was approximately twice that at 8 weeks, differing significantly across ages (Table 3). The NFL loline alkaloid was the most abundant in shoots across all ages, constituting 84–92% of total loline alkaloids. The presence of the different types of lolines increased with age, with NFL being the only loline alkaloid present in eight-week-old grass. NFL and NANL were present in 12- and 16-week samples, while by 20 weeks, all three lolines were present. In roots, only the NFL was detected, and this occurred only at 16 weeks (Table 3).

The loline alkaloid concentrations recorded in experiment 2 were higher compared to experiment 1. The three loline alkaloids (NANL, NAL, and NFL) were all present in the shoots across all plant ages except at eight weeks, when only NFL was present (Table 4). The NFL remained the dominant loline alkaloid, with NAL present at the next highest levels, while NANL occurred in the smallest quantities.

Total loline alkaloid concentrations followed a similar age-related trend, increasing markedly with plant age except between 12 and 16 weeks, where no significant differences were recorded. At 20 weeks, total loline alkaloid concentrations were nearly five times higher than those at eight weeks, while concentrations at 12 and 16 weeks were thrice higher than those at eight weeks. In root tissue, the loline profile matched that reported in experiment 1, with NFL as the only loline alkaloid detected. Unlike experiment 1, however, lolines were detected in E+ roots at every sampling age. Concentrations were comparable at 12, 16, and 20 weeks, whereas eight-week-old plants had significantly lower levels (Table 4). No lolines were present in E− root samples at any age.

#### 2.4.2. Total Flavonoid Content and Total Phenol Content

Total flavonoid content (TFC) and total phenolic content (TPC) declined in both treatments as the plant matured. with no significant differences recorded between eight and 12 weeks and between 16 and 20 weeks for both treatments. There was no treatment effect when similar ages were compared between the treatments. (Table 5). In root extracts, TFC patterns differed between grasses with endophyte and grasses without endophyte. In E+ roots, total flavonoid content was significantly greater at 20 weeks than at 8, 12, or 16 weeks, whereas TFC did not differ significantly among the younger sampling ages. In contrast. In the roots of E− grass, TFC was significantly higher at eight weeks when compared to 12 and 16 weeks, but no differences were recorded at 20 weeks.

TPC in shoots of both grasses with endophyte and grasses without endophyte also declined with age. However, shoot extracts from grasses without endophyte consistently contained significantly higher TPC than those from grasses with endophyte at all ages except at eight weeks, where the difference was not significant. In roots, TPC showed the opposite age pattern to shoots as levels of TPC reduced as the plant matured in both treatments. Extracts obtained from roots aged 16 and 20 weeks had lower TPC compared to 12 and 8-week-old grasses. In E+ roots, TPC declined up to 16 weeks and then showed a slight, non-significant increase at 20 weeks. When comparing root extracts from grasses with and without endophyte at the same age, significant differences were only at 8 and 20 weeks (Table 5).

Biomass from shoots was negatively correlated to flavonoid content (R = −0.94), indicating that increases in biomass corresponded to lower TFC levels. Phenolic content showed the same inverse relationship, with negative correlations R = −0.67 and R = −0.79 for shoots and roots, respectively.

### 2.5. Shoot and Root Weight Biomass

Fresh biomass weight of shoots and roots increased as plants matured in both treatments in experiment 1. In contrast, shoot biomass did not differ significantly across plant ages in experiment 2 for either E+ or E− plants. Although biomass was generally similar between E+ and E− plants of the same age, several significant differences were observed at specific time points, particularly in root biomass (Table 6).

Comparisons between experiments showed that shoot biomass at 8 and 12 weeks was lower in experiment 1 than in experiment 2 for both E+ and E− grasses. The shoot and root biomass at 20 weeks was, however, higher in the first experiment than in the second, indicating stronger late-season growth under the conditions of experiment 1.

Across all sampling ages, biomass from shoots was negatively correlated with TFC (R = −0.94, *p* < 0.001). Phenol content also declined with increasing biomass, showing negative correlations with biomass from roots (R = −0.79, *p* < 0.001) and shoot biomass (R = −0.67, *p* < 0.005). Together, these associations indicate that as plants matured and accumulated more biomass, phenolic and flavonoid concentrations decreased.

## 3. Discussion

Findings from this study show that extracts from shoots and roots of *Festulolium loliaceum*, in the presence or absence of the endophyte, influenced the mobility and survival of *T. primitivus*. Across experiments, extract concentration and plant age consistently explained variation in nematode immobility, whereas endophyte status modified these effects in a tissue and age-dependent manner. The responses ranged from nematostatic to nematicidal, where temporary immobilisation followed by recovery was considered nematostatic, and immobilization accompanied by measurable mortality (indicated by LD_50_ values) was considered nematicidal. Extracts from E− grasses were predominantly nematostatic, consistent with their high LD_50_ values that exceeded the concentration ranges tested, whereas extracts from E+ grasses were largely nematicidal, with LD_50_ values falling within the tested concentration range.

The influence of endophytes on nematode suppression is known to be context-dependent and varies among nematode species [12]. In host status studies, fewer *Pratylenchus scribneri* were recovered from E+ grasses [13], while no differences were observed between E+ and E− grasses for *Meloidogyne incognita* [5]. Extract-based studies show similar variability. For example, methanolic root extracts from 22-week-old tall fescue (Jesup) reduced the motility of *P. scribneri* more strongly in E+ than in E− plants across a wide concentration range and nematodes recovered after transfer to water, corresponding to a nematostatic effect [13]. These findings collectively highlight that endophyte-mediated nematode suppression cannot be generalised and varies depending on nematode species, tissue type, and class and concentration of metabolites they are exposed to.

In the present study, extract concentration and plant age were the strongest explanatory variables across all combinations tested. Extracts from younger shoots (E+ and E−) caused higher nematode immobility and produced lower LD_50_ values, while E+ root extracts became more potent as plants aged. The classification of compounds as nematicidal or nematostatic is a spectrum that is defined by factors such as exposure time and concentration of compound. At lower concentrations, compounds may be nematostatic, but with an increase in exposure time, they may cause a nematicidal effect. Similarly, at higher concentrations and short exposure time, compounds may be classified nematostatic even with the potential to have a nematicidal effect [30]. This pattern was evident here, where immobility observed at lower doses was reversible, as reflected by higher LD_50_ values. The phytochemical profile of the extracts provides insight into these patterns. No loline alkaloids were detected in E− roots or shoots, consistent with the absence of the endophyte and with previous findings that Festulolium hybrids require the endophyte for loline production [4,5]. In E+ grasses, loline profiles followed expected tissue distributions: all three lolines (NFL, NAL, NANL) were detected in shoots, whereas only NFL was detected in roots, and concentrations were generally low. The loline alkaloid NFL was present in both tissues, consistent with its status as the most abundant and widely distributed loline alkaloid across grass organs [8]. Although loline alkaloid concentrations increased with plant age, they did not correlate with nematode immobility or mortality, indicating that lolines were unlikely to be the main/only contributors to the bioactivity observed. Consistent findings were reported in earlier assays, where lolines in extracts obtained from E+ grass did not affect *M. incognita* motility relative to E− extracts, albeit under conditions involving younger plants and reduced extract concentrations [5]. The observation that E− shoots in the present study also produced marked bioactivity, despite lacking detectable lolines, further supports the involvement of additional metabolites.

Age-structured patterns of total phenolic and flavonoid compounds provided a more parsimonious explanation for the observed bioactivity. Strong negative correlations were found between shoot biomass and both flavonoid and phenolic concentrations. As plants matured and accumulated greater biomass, concentrations of these antioxidant compounds declined. Similar age-related declines in phenolics have been reported in sorghum [31], and in residues from younger tree bark relative to those from older bark [32].

The negative correlations between shoot biomass and the measured antioxidants (total phenols and flavonoids) can be interpreted in three complementary ways. First, they are consistent with a growth-dilution effect, in which concentrations of secondary metabolites decline because structural biomass accumulates more rapidly than defence metabolites, even if the absolute quantities of these compounds do not decrease [33]. Second, they may reflect ontogenetic reprogramming of metabolism, as phenolic profiles and total phenolic or antioxidant levels often vary substantially with developmental stage, with younger tissues typically exhibiting higher concentrations than mature tissues [34]. Third, the pattern aligns with the broader growth secondary metabolism trade-off framework, in which investment in growth and primary metabolism can occur at the expense of secondary or defence compounds (including phenolics), depending on developmental and resource conditions [35].

Younger shoots contained higher totals of phenolics and flavonoids than older shoots, and these pools covaried with lower LD_50_ values and higher immobility in *T. primitivus.* Although this study did not directly evaluate purified phenolics or flavonoids against *T. primitivus*, several studies have shown that these compounds are bioactive against other plant-parasitic nematodes. For example, the polyphenol chlorogenic acid caused reversible immobilisation of *P. scribneri* [13]. In addition, *o*-coumaric acid, syringic acid, and caffeic acid were effective against *M. javanica* at 15 µg mL^−1^ [14]. Compounds such as methyl 4-hydroxycinnamate and methyl 4-hydroxybenzoate obtained from *Allium grayi* showed activity against *M. incognita* [36], while salicylic acid displayed direct toxicity to *M. incognita* and reduced gall formation in tomato [37]. Phenols have also been reported to suppress *P. penetrans* egg hatching. [38].

Flavonoids exhibit comparable activity. After exposure to 5–25 µg mL^−1^ of coumestrol, the motility of *P. scribneri* was inhibited; however, no effect was observed towards *M. javanica* [22], and medicarpin inhibited the motility of *P. penetrans* in vitro [16]. The widely distributed flavonoid quercetin, as a soil drench, caused a decline in *M. javanica* reproduction [39]. Collectively, these findings support the view that phenolic and flavonoid compounds can contribute meaningfully to nematode suppression and may help explain the patterns recorded in the study.

The absence of a correlation between lolines concentrations and bioactivity across plant ages, together with the consistently greater potency of E+ shoot extracts compared with E− shoots and the clear age-dependent trends that paralleled shifts in phenolic and flavonoid levels, suggests that the bioactivity is most likely driven by additive and/or synergistic effects of multiple compounds. This interpretation aligns with previous findings in tall fescue, where nematotoxic effects against *P. scribneri* were attributed to additive contributions from polyphenolics and ergot alkaloids rather than from a single dominant metabolite [13].

In conclusion, these results indicate that (i) E+ shoot extracts are more potent than E− shoots based on LD_50_ values, although this difference is unlikely to be attributable to loline alkaloids alone; (ii) root extracts predominantly exert nematostatic rather than nematicidal effects under the conditions tested; and (iii) age-linked shifts in phenolic and flavonoid pools represent a plausible mechanism underlying the mortality patterns observed in *T. primitivus*. Because this study did not test purified lolines or individual phenolic or flavonoid compounds directly against *T. primitivus*, interpretations regarding the roles of these chemical classes remain correlative.

## 4. Conclusions

Methanolic shoot and root extracts of *Festulolium loliaceum* affected the mobility and survival of *Trichodorus primitivus*, with extract concentration and plant age explaining most variation across experiments. Endophyte status further modified these responses in a tissue and age-dependent manner, with E+ shoot extracts showing greater nematicidal activity than E− shoots, while root extracts were largely nematostatic. Loline alkaloids, detected only in E+ tissues and increasing with plant age, did not correlate with immobility or LD_50_. In contrast, age-related shifts in total phenolics and flavonoids aligned with stronger effects in younger shoots, supporting a multi-compound model of activity. Although purified metabolites were not tested, the findings identify plant age, tissue type, and endophyte status as key determinants of extract bioactivity against *T. primitivus*.

## 5. Materials and Methods

### 5.1. Glasshouse Growth Conditions and Experimental Design

Seeds of E+ and E− *Festulolium loliaceum* grass were provided by Cropmark Seeds Ltd., Christchurch, New Zealand. The seeds were sown into pots (20 cm diameter × 15 cm depth) filled with John Innes No. 2 compost at a depth of 2 cm, in accordance with the supplier’s recommendation. A sowing rate of 25 kg ha^−1^ was used, which—after scaling to the pot surface area (314 cm^2^) relative to 1 ha (100,000,000 cm^2^)—corresponded to approximately 0.08 g of seed per pot.

Two glasshouse experiments were run (December 2022–May 2023; February–June 2023). Shoots and roots were sampled destructively at four plant ages (8, 12, 16, and 20 weeks after sowing). Glasshouse conditions were maintained under a 16 h photoperiod. In Experiment 1, mean day/night temperatures were 24/8 °C with ~67% relative humidity, whereas in Experiment 2, mean day/night temperatures were 28/5 °C with ~62% relative humidity. Wuxal liquid fertiliser (8–3.5–5 NPK; Aglukon Spezialdünger GmbH, Düsseldorf, Germany) was applied at planting, and plants were watered twice weekly. The swards were not clipped during the duration of the experiment.

The experiment was laid out in a randomised complete block design. Three pots were allocated as replicates for each combination of endophyte status and harvest age.

### 5.2. Endophyte Status (Epichloë uncinata) Assessment

Endophyte status was verified using a commercial tiller tissue-print immunoblot system (*Epichloë* Endophyte Tissue Print Immunoblot Tiller Kit; Cropmark Seeds Ltd., Christchurch, New Zealand). At 8 weeks post-sowing, ten tillers were sampled from each treatment (with endophyte, E+; without endophyte, E−), with tillers selected at random from across replicate pots. The assay provides a nitrocellulose sheet and all components required for immunodetection, including a monoclonal primary antibody, an alkaline phosphatase-conjugated secondary antibody, a chromogenic reagent, and blocking solutions (skim milk and Tris/NaCl).

Tillers were trimmed from the basal section of the stem to produce a clean, level cross-section; any damaged, necrotic, or diseased tissue was removed prior to printing. The freshly exposed tiller base was then applied to the nitrocellulose membrane for 3–4 s to generate the tissue imprint.

Membranes were blocked in 10 mL blocking buffer within a Petri dish on an orbital shaker (60 rpm) for 30 min, after which the buffer was discarded. The membrane was then incubated with the primary antibody for 1 h with gentle agitation, followed by two washes in fresh blocking solution (10 mL; 5 min each). The secondary antibody (alkaline phosphatase-linked) was applied for a further 1 h under agitation and removed by two additional 5 min washes using fresh blocking buffer.

Blot development was initiated by adding chromogen, immediately covering the dish with aluminium foil to exclude light, and shaking for 30 min. Colour formation was checked every 15 min. After development, chromogen was discarded, and the membrane was washed repeatedly with cold tap water. Imprints were examined using a Leica M80 stereomicroscope (Leica Microsystems (Schweiz) AG, Heerbrugg, Switzerland) at 20× magnification., and endophyte presence/absence was scored, following the manufacturer’s guidelines based on staining the tiller print.

### 5.3. Stubby Root Nematodes Sampling

Stubby root nematodes used in the experiments were obtained from soil sampled at a long-established SRN-infested field site at Harper Adams University (Crabtree Leasow). The SRN was extracted using the Seinhorst two-flask extraction procedure [40]. The species used for the assessments was confirmed as *T. primitivus.*

### 5.4. Plant Extracts Preparation and Assay Design

Shoots and root material were sampled from E+ and E− grasses at 8, 12, 16, and 20 weeks after sowing. At each time interval, three treatment pots were destructively sampled. Fresh biomass obtained from shoots and roots was measured, after which tissues were freeze-dried for seven days (GVD6/13 MKI freeze dryer, GIROVAC Ltd., North Walsham, UK), ground to a fine powder, and sieved to 1 mm. For each plant age × endophyte status × tissue type, dried material from the three pots was pooled to form a composite sample for extraction.

Crude methanolic extracts were prepared by combining 4 g of freeze-dried, ground tissue with 40 mL of 98% methanol and shaking at 100 rpm for 20 h (HS 501 digital, IKA, Staufen, Germany). Filtration was carried out using Whatman No. 1 paper, after which the filtrates were collected in pre-weighed containers. Methanol was removed by rotary evaporation, and the remaining crude extract was reconstituted in sterile distilled water and mixed until completely solubilised. The reconstituted solutions were then sterilised by sequential syringe filtration through 0.45 µm followed by 0.20 µm membranes. Stock concentrations were determined from the mass of dried crude extract obtained after solvent removal, and dilutions were expressed as µg mL^−1^ (*w*/*v*) of dried crude extract in sterile distilled water.

For each combination of plant age, endophyte status, and tissue (shoot or root), five test concentrations were prepared (5000, 2500, 1250, 625, and 312.5 µg mL^−1^). Sterile distilled water was included as the negative control. The in vitro bioassays were conducted in 5 mL glass tubes using an exposure volume of 2 mL per replicate, and tubes were incubated at 20 ± 2 °C under dark conditions. Each age × endophyte status × concentration combination was replicated five times for both shoots and root extracts, and the experiment was performed twice as independent runs. For each replicate, 20 mixed-stage stubby root nematodes were transferred into the tubes and nematode mobility assessed in a repeated measures design after 24, 48, and 72 h by gentle mechanical stimulation using an eyelash needle.

Individual nematodes were scored as immobile if no movement was observed following gentle stimulation with an eyelash needle; such immediate immobility was recorded as a nematostatic response unless immobility persisted after the recovery period. After the 72 h exposure, nematodes were transferred to distilled water for a 48 h recovery period, after which mobility was reassessed. Mortality (nematicidal effect) was defined as irreversible immobility following the 48 h recovery and recorded as dead/total for each replicate. Observations were made under the same assay conditions.

### 5.5. Quantification of Phytochemicals

#### 5.5.1. Total Flavonoid Content (TFC)

Total flavonoids in shoot and root extracts were quantified following the aluminium chloride (AlCl_3_) colourimetry method [41]. Quercetin served as the external standard (0–200 µg mL^−1^). Samples were prepared at 5000 µg mL^−1^, and 500 µL aliquots were mixed with 250 µL AlCl_3_ reagent (50 g L^−1^ in methanol) and 4.25 mL methanol. After centrifugation (1300× *g*, 2 min), the reaction mixtures were left for 30 min, and the absorbance was measured at 510 nm. Methanol served as the blank. Flavonoid concentrations were determined from the quercetin calibration curve (y = 0.007x − 0.006, R^2^ = 0.97) and expressed on a quercetin basis (µg QE g^−1^ dry material). The limit of detection was 287 µg mL^−1^.

#### 5.5.2. Total Phenolic Content (TPC)

Total phenolics in shoot and root extracts were quantified using the Folin–Ciocalteu assay [42]. A gallic acid standard series (0–250 µg mL^−1^) was used for quantification. Extract solutions were prepared at 5000 µg mL^−1^. For analysis, 250 µL of extract was added to 250 µL of Folin–Ciocalteu reagent pre-diluted 1:1 (*v*/*v*) with water, then 500 µL of saturated Na_2_CO_3_ and 4 mL distilled water were added. The reaction mixtures were kept in the dark for 25 min before centrifugation (3000× *g*, 10 min). Absorbance was measured at 725 nm. Total phenolic concentrations were calculated from the gallic acid calibration equation (y = 0.085x − 0.066, R^2^ = 0.98) and reported as µg gallic acid equivalents per g dry material (µg GAE g^−1^). The limit of detection was 77.6 µg mL^−1^.

#### 5.5.3. Loline Alkaloids Concentrations 

Loline alkaloid quantification followed a modified protocol from [4,43]. Briefly, 250 mg of freeze-dried tissue was weighed into a 6 mL glass vial and combined with 5 mL of a 95:5 (*v*/*v*) dichloromethane: ethanol solvent mixture plus 250 µL of saturated sodium bicarbonate. Samples were shaken at 200 rpm for 1 h. An internal standard, 4-phenomorpholine (60 µg mL^−1^; Sigma Aldrich^®^, Sydney, Australia), was used to spike the solvent used for extraction. The resulting extracts were filtered through a cotton-plugged pipette tip, then 1 mL of the filtrate was aliquoted into a 2 mL GC vial.

Loline alkaloids were analysed by gas chromatography on a Shimadzu GC-2010 fitted with a flame ionisation detector and a ZB-5 capillary column (30 m × 0.25 mm × 0.25 µm; Phenomenex^®^, Auckland, New Zealand). Hydrogen served as the carrier gas (6 mL min^−1^). Detector flow rates were 40 mL min^−1^ for H_2_ and 400 mL min^−1^ for air. The oven temperature programme ramped from 40 °C to 320 °C at 20 °C min^−1^ and was held at 320 °C for 5 min. Injections were made in splitless mode (1 µL). Target compounds were identified by retention time: *N*-methylloline (5.9 min), 4-phenomorpholine (6.9 min), *N*-acetylnorloline (8.2 min), *N*-formylloline (8.4 min), and *N*-acetylloline (8.7 min). Quantification was based on calibration curves prepared using loline standards purified from Barrier U2™ seed (Cropmark Seeds Ltd., Christchurch) and from *Festulolium* containing *E. uncinata* [5]. The limit of detection for loline alkaloids was 30 µg g^−1^.

## 6. Statistical Analysis

Data were analysed in RStudio using R version 4.5.1 [44]. Homogeneity of variance between trials was assessed using Levene’s test. Because the two trials showed significant differences (*p* < 0.05), the data were analysed separately. Immobility data (24, 48, 72 h) were modelled using mixed-effects beta regression (package glmmTMB), with fixed factors age, treatment, concentration, and random factor time. Pairwise contrasts (*p* < 0.05) between E+ and E− grass at each plant age were generated using emmeans.

Mortality was analysed at each plant age (8, 12, 16, 20 weeks) and for each treatment (E+ vs. E−). For each subset, a log-logistic dose response model (two-parameter log-logistic, LL.2(), was fitted in RStudio using the drc package (drm ()), treating mortality as a binomial response (dead/total) and using the corresponding sample sizes as weights The LD_50_ was then estimated as the lethal dose causing 50% mortality from the fitted curve using ED (model, 50, interval = “delta”), which also provided the associated standard error and 95% lower and upper confidence limits [45].

Correlation coefficients were computed using the ggpubr package on RStudio. Data on shoot biomass, loline alkaloids, and antioxidants (TPC and TFC) were analysed using Anova and means separated using Tukey’s HSD at *p* < 0.05.

## Figures and Tables

**Table 1:** Experiment 1: Median lethal dose (LD_50_, µg mL^−1^), standard error, and 95% confidence limits for *Festulolium loliaceum* shoot and root extracts from plants with and without endophyte at different plant ages against the stubby root nematode *Trichodorus primitivus*.

Plant Age			Shoots			Roots		
Grass with Endophyte (E+)	LD_50_	SE	Lower	Upper	LD_50_	SE	Lower	Upper
8 weeks	627 a	94	442	812	nd	nd	nd	nd
12 weeks	1520 **a**	151	1222	1819	6274 **b**	3063	189	12,360
16 weeks	7424 **b**	3017	1471	13,376	3399 **a**	343	2717	4081
20 weeks	4275 **b**	430	3425.83	5124	2847 **a**	374	2104	3590
Grass without endophyte (E−)	LD_50_	SE	Lower	Upper	LD_50_	SE	Lower	Upper
8 weeks	860 **a**	97	669	1051	nd	nd	nd	nd
12 weeks	4717 **b**	389	3949	5485	5675 **b**	711	4263	7087
16 weeks	9488 **c**	543	8402	10,574	6471 **b**	1169	4149	8792
20 weeks	73,450 **d**	3318	66,815	80,086	140,093 **c**	8037	124,018	156,166

LD_50_ = median lethal dose causing 50% mortality; Upper 95% CI/Lower 95% CI = upper and lower 95% confidence limits; nd = no mortality recorded; SE = standard error; Within each tissue set (shoots and roots), different lowercase letters indicate significant differences in LD_50_ values among ages and endophyte status (E+ versus E−) based on Tukey’s HSD post hoc test (*p* < 0.05).

**Table 2:** Experiment 2: Median lethal dose (LD_50_, µg mL^−1^), standard error, and 95% confidence limits for *Festulolium loliaceum* shoot and root extracts from plants with and without endophyte at different plant ages against the stubby root nematode *Trichodorus primitivus*.

Plant Age	Shoots				Roots			
Grass with endophyte (E+)	LD_50_	SE	Lower	Upper	LD_50_	SE	Lower	Upper
8 weeks	1411 **a**	522	381	2440	nd	nd	nd	nd
12 weeks	1778 **a**	281	1127	2338	6471 **a**	1169	4149	8792
16 weeks	3060 **b**	716	1647	4472	5404 **a**	622	4169	6639
20 weeks	2534 **b**	436	1672	3396	3399 **b**	343	2717	4081
Grass without endophyte (E−)	LD_50_	SE	Lower	Upper	LD_50_	SE	Lower	Upper
8 weeks	1171 **a**	185	806	1536	nd	nd	nd	nd
12 weeks	2717 **b**	439	1850	3584	8661 **a**	2631	3434	13,888
16 weeks	7709 **c**	2543	2624	12,794	12,345 **c**	6027	373	24,317
20 weeks	19,967 **d**	5000	9967	29,967	21,243 **d**	20,007	18,498	60,985

LD_50_ = median lethal dose causing 50% mortality; Upper 95% CI/Lower 95% CI = upper and lower 95% confidence limits; nd = no mortality recorded; SE = standard error; Within each tissue set (shoots and roots), different lowercase letters indicate significant differences in LD_50_ values among ages and endophyte status (E+ versus E−) based on Tukey’s HSD post hoc test (*p* < 0.05).

**Table 3:** Experiment 1: Concentrations of loline alkaloids in shoots and roots of *Festulolium loliaceum* (dry matter, DM) with endophyte and without endophyte at different plant ages.

Plant Age	Shoots				Roots			
Grass with endophyte (E+)	NFL	NAL	NANL	Total lolines	NFL	NAL	NANL	Total lolines
8 weeks	1011	nd	nd	1011 **a**	nd	nd	nd	nd
12 weeks	1420	254	nd	1674 **b**	nd	nd	nd	nd
16 weeks	1623	145	nd	1768 **c**	144	nd	nd	144
20 weeks	2144	94	98	2336 **d**	nd	nd	nd	nd
Grass without endophyte (E−)	NFL	NAL	NANL	Total lolines	NFL	NAL	NANL	Total lolines
8 weeks	nd	nd	nd	nd	nd	nd	nd	nd
12 weeks	nd	nd	nd	nd	nd	nd	nd	nd
16 weeks	nd	nd	nd	nd	nd	nd	nd	nd
20 weeks	nd	nd	nd	nd	nd	nd	nd	nd

Grass with endophyte (E+), grass without endophyte (E−); DM = dry matter; concentrations are expressed as µg g^−1^ DM (micrograms per gram of dry matter); NFL = N-formylloline; NAL = N-acetylloline; NANL = N-acetylnorloline. Total loline = NFL + NAL + NANL; nd = no lolines detected (<30 µg g^−1^); Total loline alkaloids means in paired columns (E+ and E−) followed by different lowercase letters differ significantly based on Tukey’s HSD post hoc test (*p* < 0.05).

**Table 4:** Experiment 2: Concentrations of loline alkaloids in shoots and roots of *Festulolium loliaceum* (dry matter, DM) with endophyte and without endophyte at different plant ages.

Plant Age	Shoots				Roots			
Grass with endophyte (E+)	NFL	NAL	NANL	Total lolines	NFL	NAL	NANL	Total lolines
8 weeks	1449	nd	nd	1449 **a**	93	nd	nd	93 **a**
12 weeks	3716	408	162	4286 **ab**	247	nd	nd	247 **b**
16 weeks	3682	405	172	4258 **ab**	130	nd	nd	130 **ab**
20 weeks	5616	490	368	6475 **b**	111	nd	nd	111 **ab**
Grass without endophyte (E−)	NFL	NAL	NANL	Total lolines	NFL	NAL	NANL	Total lolines
8 weeks	nd	nd	nd	nd	nd	nd	nd	nd
12 weeks	nd	nd	nd	nd	nd	nd	nd	nd
16 weeks	nd	nd	nd	nd	nd	nd	nd	nd
20 weeks	nd	nd	nd	nd	nd	nd	nd	nd

Grass with endophyte (E+), grass without endophyte (E−); DM = dry matter; concentrations are expressed as µg g^−1^ DM (micrograms per gram of dry matter); NFL = N-formylloline; NAL = N-acetylloline; NANL = N-acetylnorloline. Total loline = NFL + NAL + NANL; nd = no lolines detected (<30 µg g^−1^); Total loline alkaloids means in paired columns (E+ and E−) followed by different lowercase letters differ significantly based on Tukey’s HSD post hoc test (*p* < 0.05).

**Table 5:** Total flavonoid content (TFC) and total phenolic content (TPC) in methanolic shoot and root extracts of *Festulolium loliaceum* plants with endophyte and without endophyte at different plant ages.

Total Flavonoid Content (µg QE g^−1^)					Total Phenol Content (µg GAE g^−1^)			
	Shoots		Roots		Shoots		Roots	
Plant Age	E+	E−	E+	E−	E+	E−	E+	E−
8 weeks	441 **a**	438 **a**	436 **a**	447 **a**	1262 **ab**	1346 **a**	1428 **a**	1607 **b**
12 weeks	426 **a**	431 **a**	429 **a**	409 **b**	1060 **c**	1239 **b**	1370 **a**	1224 **a**
16 weeks	414 **b**	412 **b**	412 **b**	416 **b**	853 **d**	1022 **c**	834 **c**	665 **c**
20 weeks	409 **b**	407 **b**	471 **c**	421 **ab**	956 **cd**	1013 **c**	1256 **a**	692 **c**

Grass with endophyte (E+), grass without endophyte (E−). Values are means (n = 3) and are expressed on a dry-weight basis (µg g−1 DW). TFC is expressed as quercetin equivalents (QE), and TPC as gallic acid equivalents (GAE). Within each tissue set (shoot TFC, root TFC, shoot TPC, and root TPC), different lowercase letters indicate significant differences among ages and endophyte status (E+ versus E−) based on Tukey’s HSD post hoc test (*p* < 0.05).

**Table 6:** Mean fresh root and shoot biomass (g) of *Festulolium loliaceum* grown under glasshouse conditions with (E+) and without (E−) endophyte across two independent experiments.

Experiment 1					Experiment 2			
	Shoots		Roots		Shoots		Roots	
Plant Age	E+	E−	E+	E−	E+	E−	E+	E−
8 weeks	42 **a**	48 **a**	16 **a**	36 **a**	323 **a**	398 **a**	37 **a**	54 **a**
12 weeks	125 **b**	157 **b**	66 **a**	107 **b**	457 **a**	342 **a**	127 **b**	71 **a**
16 weeks	224 **bc**	353 **c**	88 **ab**	149 **b**	395 **a**	348 **a**	198 **b**	94 **ab**
20 weeks	602 **d**	695 **d**	115 **b**	143 **b**	244 **a**	377 **a**	70 **ab**	84 **a**

Grass with endophyte (E+), grass without endophyte (E−). Values are means (n = 3). Experiments 1 and 2 are analysed separately, and shoots and roots are analysed separately. Within each experiment × tissue set (shoots or roots), different lowercase letters denote significant differences among the eight means (E+ and E− across four ages) based on Tukey’s HSD post hoc test (*p* < 0.05).

## Data Availability

The original contributions presented in this study are included in the article. Further inquiries can be directed to the corresponding author(s).
